# The association of neighborhood-level deprivation with glioblastoma outcomes: a single center cohort study

**DOI:** 10.1007/s11060-025-05002-3

**Published:** 2025-04-07

**Authors:** Yifei Sun, Dagoberto Estevez-Ordonez, Travis J. Atchley, Burt Nabors, James M. Markert

**Affiliations:** 1 Marnix E. Heersink School of Medicine, University of Alabama at Birmingham, Birmingham, AL USA; 2 Department of Neurosurgery, University of Alabama at Birmingham, Birmingham, AL USA; 3 O’Neal Comprehensive Cancer Center, University of Alabama at Birmingham, Birmingham, AL USA; 4 Division of Neuro-Oncology, University of Alabama at Birmingham, Birmingham, AL USA

**Keywords:** Area deprivation, Glioblastoma, Neighborhood socioeconomic status, Outcomes, Survival

## Abstract

Glioblastoma is the most common primary brain malignancy. Though literature has suggested the association of glioblastoma outcomes and socioeconomic status, there is limited evidence regarding the association of neighborhood-level socioeconomic deprivation on glioblastoma outcomes. The aim of this study was to assess the impact of neighborhood-level socioeconomic deprivation on glioblastoma survival. We retrospectively reviewed all adult glioblastoma patients seen at a single institution from 2008 to 2023. Neighborhood deprivation was assessed via Area Deprivation Index (ADI), with higher ADI indicating greater neighborhood socioeconomic disadvantage. Log-rank tests and multivariate cox regression was used to assess the effect of ADI and other socioeconomic variables while controlling for a priori selected clinical variables with known relevance to survival. In total, 1464 patients met inclusion criteria. The average age at diagnosis was 60 ± 14 years with a median overall survival of 13.8 months (IQR 13-14.8). The median ADI of the cohort was 66(IQR 46–84). Patients with high neighborhood disadvantage had worse overall survival compared to patients with those without (11.7 vs. 14.8 months, *p* =.001). In the multivariable model, patients with high neighborhood disadvantage had worse overall survival (HR 1.25, 95%CI 1.09–1.43). To account for changes in WHO guidelines, we implemented the model on patients diagnosed between 2017 and 2023 and findings were consistent (HR 1.26,95%CI 1.01–1.56). We report the first study demonstrating glioblastoma patients with higher neighborhood deprivation have worse survival after controlling for other socioeconomic and biomolecular markers. Neighborhood socioeconomic status may be a prognostic marker for glioblastoma survival.

**Supplementary Information:**

The online version contains supplementary material available at 10.1007/s11060-025-05002-3.

## Introduction

Glioblastoma is the most common primary brain malignancy, comprising around half of all primary brain tumors [1]. Despite recent progress in treatments, prognosis for patients remain poor, with median survival around 15 months [2]. Thus, it is of interest to better understand the risk factors that are associated with worsened survival. Recent literature has identified a connection between socioeconomic disparity and worsened outcomes in patients with glioblastoma [3, 4].

Socioeconomic factors are complex and can differentially affect outcomes. Recent studies have identified racial and income-related disparities in surgical care, chemotherapy, and radiotherapy for patients in glioblastoma [4, 5]. Other studies have also identified disparities in glioblastoma outcomes on the basis of race, insurance status, age, and educational status, as well as inequalities in timeliness of care as well [6].

However, the effect of neighborhood-level SES status on glioblastoma survival remains unclear. Much of socioeconomic literature data at the ZIP code level, which has been shown to be poor proxies of socioeconomic status [7]. Furthermore, currently commonly utilized measures of SES are often poorly generalizable and subject to regional bias [8, 9]. Area Deprivation Index (ADI) is a tool developed by the Health Resources and Services Administration (HRSA) that measures neighborhood-level disadvantage by taking into account 17 measures of socioeconomic disparity in 4 main domains: education, income/employment, housing, and household characteristics [7]. These variables are then integrated into a standardized national ranking from 0 to 100, with 100 being the highest level of deprivation.

Amongst the most studied area-level measures of socioeconomic disadvantage and independently validated by studies across many domains of health outcomes research, neighborhood deprivation has been used to link socioeconomic disparity to poor patient outcomes in diabetes research, cardiovascular research, and other surgical specialties [10–12]. Neighborhood deprivation has also gained increased attention due its inclusion in incentives programs and reimbursement adjustment calculations by the Center for Medicare/Medicaid (CMS) [13].

Despite its widespread adoption in other fields, there is little evidence regarding the association of neighborhood-level socioeconomic status on the overall survival (OS) of patients with glioblastoma. There is also little literature that examine the effect of these socioeconomic factors on glioblastoma outcomes in the context of clinically important molecular markers such as MGMT methylation and IDH wild-type (WT) status.

The aim of this study was to assess the impact of neighborhood-level socioeconomic disadvantage on glioblastoma survival in the largest cohort to date and to better understand how socioeconomic status affects outcomes for patients with glioblastoma. To our knowledge, this is the first report to describe the association of neighborhood level socioeconomic deprivation with OS in glioblastoma.

## Methods

We performed a single center retrospective review with approval from the Institutional Review Board (IRB- 300011516). This manuscript was written in compliance with STROBE (Strengthening the Reporting of Observational Studies in Epidemiology) [14].

### Participants and data collection

We retrospectively identified all adult patients, 18 years or older, with histopathological new glioblastoma diagnosis seen at our institution between January 1st, 2008 and December 31st, 2023. In total, 1464 patients met inclusion criteria. The electronic medical record (EMR) was reviewed for variables on patient demographics, socioeconomic background, geography, and treatment characteristics. Due to the retrospective nature of this study, patient consent was not needed.

### Defining variables

Variables were defined a priori with advice from the senior authors. Study variables included were age at diagnosis, race, gender, marital status, extent of surgical resection, IDH status MGMT methylation status, history of chemotherapy and history of radiotherapy. Patient addresses were extracted from the EMR and were geocoded using ArcGIS software. Federal Information Processing System (FIPS) codes were extracted and correlated to its individual Area Deprivation Index (ADI), with higher ADI relating to more socioeconomic deprivation. ADI was retrieved from the Neighborhood Atlas dataset produced by the Center for Health Disparities Research at the University of Wisconsin School of Medicine and Public Health.^4^ Patients with high neighborhood disadvantage were identified as patients in the top national quartile ranking of ADI (> 75) according to previous literature [15]. Elixhauser Comorbidity scores were abstracted from ICD 9/10-CM codes and calculated with Van Walraven weighting [16].

### Statistical analysis

Univariable analysis including Student’s t-test, one-way analysis of variance (ANOVA), Chi squared test, and Wilcoxon rank sum test were used to compare incidences of chemotherapy, radiotherapy extent of resection, and other demographic variables between patients with high and low neighborhood disadvantage. Kaplan Meier curves were used to investigate differences in survival between groups of interest, and log rank tests were used to assess differences in survival.

**Fig. 1:**
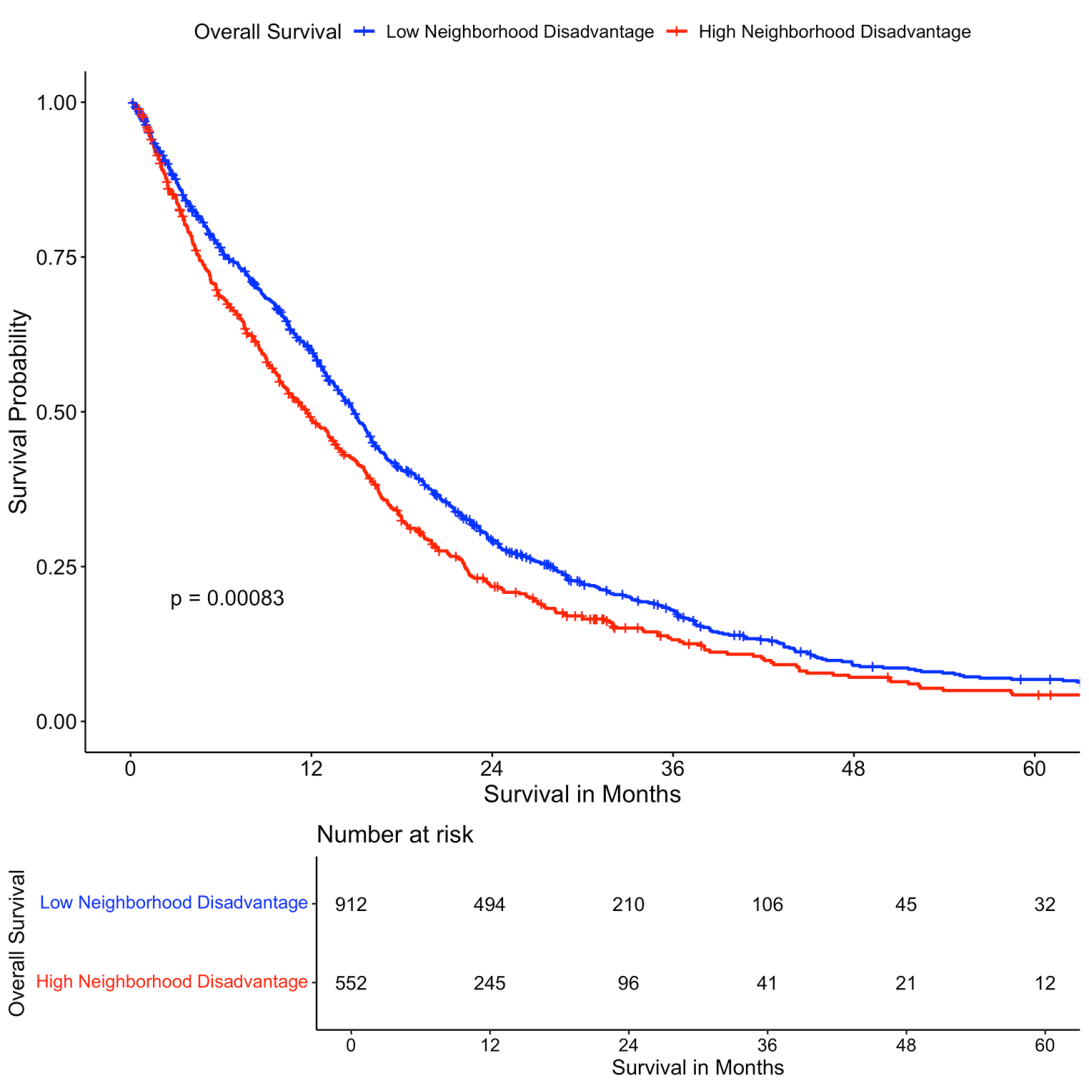
Kaplan Meier survival plot stratified by neighborhood disadvantage

Sensitivity analysis was conducted by performing multiple methods of imputation for missing data as well as replicating the model in patients diagnosed and treated after the WHO guidelines in 2016. All statistical analyses were performed using R studio (version 4.3.1) [17]. Further details on statistical analysis can be found in the supplementary content (Supplementary Content, Tables 1, 2, 3, 4 and 5 S, Fig. 1 S).

## Results

In total, 1464 patients met inclusion criteria. The mean age at diagnosis was 60 ± 14 years. Of these patients, 155 (11%) were black and 816 (56%) were male. At time of censoring, 249 (17%) were alive. Of these patients, 671(46%) received complete resection, 1235 (84%) received radiotherapy and 1219 (83%) received chemotherapy. The median ADI was 66 (IQR 46–84). Ninety-two (6.3%) of the patients had IDH mutations and 344 (23%) of the patients were of MGMT-methylated status. The median OS (mOS) of the cohort was 13.7 months (IQR 12.99–14.16). Further details on patient demographics and characteristics can be found in the Table 1 and supplement (Supplementary Content, Table 6 S).

**Table 1:** Patient characteristics and demographics

	High neighborhood disadvantage		*p*-value^2^
	No, *N* = 912^*1*^	Yes, *N* = 552^*1*^	
Age (years)	62 (52, 70)	62 (51, 69)	0.8
< 45	124 (14%)	87 (16%)	
45–54	142 (16%)	89 (16%)	
55–64	248 (27%)	144 (26%)	
65–74	281 (31%)	164 (30%)	
≥75	117 (13%)	68 (12%)	
Sex			0.5
Female	398 (44%)	250 (45%)	
Male	514 (56%)	302 (55%)	
Race			< 0.001
Black	60 (6.6%)	95 (17%)	
Other	62 (6.8%)	23 (4.2%)	
White	790 (87%)	434 (79%)	
Married	682 (75%)	351 (64%)	< 0.001
Insurance			< 0.001
Indigent/Self Pay	28 (3.1%)	23 (4.2%)	
Medicaid	51 (5.6%)	60 (11%)	
Medicare	365 (40%)	225 (41%)	
Private	468 (51%)	244 (44%)	
Median Household Income (USD)	55,681 (47,276, 68,608)	42,116 (37,433, 48,371)	< 0.001
Vital Status			0.12
Alive	166 (18%)	83 (15%)	
Deceased	746 (82%)	469 (85%)	
IDH status			0.4
IDH-Mut	55 (6.0%)	37 (6.7%)	
IDH-WT	566 (62%)	324 (59%)	
Missing	291 (32%)	191 (35%)	
MGMT Status			0.037
Methylated	211 (23%)	133 (24%)	
Unknown	340 (37%)	236 (43%)	
Missing	361 (40%)	183 (33%)	
Extent of Resection			0.021
Biopsy	247 (27%)	183 (33%)	
Complete resection	441 (48%)	230 (42%)	
Partial Resection	224 (25%)	139 (25%)	
Received Radiotherapy	782 (86%)	453 (82%)	0.06
Received Chemotherapy	770 (84%)	449 (81%)	0.12
ADI	52 (36, 64)	87 (82, 92)	< 0.001
RUCA			< 0.001
Metropolitan	753 (83%)	309 (56%)	
Micropolitan	111 (12%)	114 (21%)	
Rural	8 (0.9%)	43 (7.8%)	
Small Town	40 (4.4%)	86 (16%)	
Distance from Institution (miles)			< 0.001
<60	426 (47%)	191 (35%)	
60–200	362 (40%)	336 (61%)	
≥200	124 (14%)	25 (4.5%)	
Elixhauser Comorbidity Score	12 (8, 20)	13 (4, 20)	0.2
Missing	92 (10%)	68 (12%)	

^*1*^ n (%); Median (IQR) ^*2*^ Pearson’s Chi-squared test; Wilcoxon rank sum test. * ADI: Area Deprivation Index, High ADI-ADI > 75, IDH: Isocitrate dehydrogenase; WT: Wild Type; Mut: Mutant; MGMT: O6-Methylguanine methyl transferase

### Univariable comparison analysis

Patients with high neighborhood disadvantage were more likely to be black (17% vs. 6.6%, *p* <.001), higher rates of Medicaid/Medicare (52% vs. 45.6%, *p* <.001), more likely to live greater than 60 miles from the institution, and more likely to live in a rural region (7.8% vs. 0.9% *p* <.001). These patients were also less likely to undergo complete resection compared to those without high neighborhood disadvantage (42% vs. 48%, *p* =.021). There was no difference in comorbidities between the two groups. The results of this analysis can be found in Table 1 and the supplement (Supplementary Content Table 7 S).

### Univariable survival comparison analysis

In univariable survival comparison, black patients had longer mOS (15.2 months, 95%CI 10.6–18.0) compared to Caucasian patients (13.5 months 95%CI 12.6–14.5). Patients who had high neighborhood disadvantage had lower mOS (11.67 months, 95% CI 13.8–15.8) compared to those who did not (14.83 months, 95%CI 10.2–13.4). In a cohort of IDH-WT only patients, we found that patients with high neighborhood disadvantage had lower mOS as well (11.1 months, 95%CI 9.53–13.0) compared to those who did not (14.1 months, 95%CI 13.1–15.5). Patients who were privately insured had longer mOS (15.4 months, 95%CI 14.5–16.5) compared to those who were publicly insured (11.5 months, 95%CI 10.0–12.9) and those who were uninsured (12.4 months, 95%CI 7.3–21.2). The results of this analysis and other survival analysis can be found in Table 2 and the supplement (Supplementary Content, Table 8 S, Fig. 3S). Kaplan Meier survival curves stratifying for neighborhood disadvntage are presented in Fig. 1.

**Table 2:** Univariable survival analysis

	Level	Median survival	CI lower	CI upper	*p*-value^1^
Age	< 45	26.27	22.03	32.12	< 0.001
	45–54	17.69	15.85	20.51	
	55–64	14.66	13.08	16.18	
	65–74	9.86	8.38	11.28	
	≥ 75	6.12	4.67	7.53	
Sex	Female	14.10	12.53	15.48	0.25
	Male	13.51	12.79	14.83	
Income Class*	Lower	13.84	13.02	15.06	0.51
	Middle	13.51	11.24	15.09	
	Upper	10.32	1.12	NA	
Neighborhood Disadvantage (ADI)**	Low	14.83	13.81	15.78	< 0.001
	High	11.67	10.22	13.38	
Insurance Category	Private	15.39	14.53	16.50	< 0.001
	Public	11.51	9.99	12.85	
	Self-Pay/Indigent	12.36	7.27	21.17	
RUCA***	Metropolitan	14.24	13.12	15.16	0.18
	Micropolitan	13.71	11.74	15.75	
	Rural	11.57	9.04	19.96	
	Small Town	11.97	9.50	15.52	
Race	Black	15.19	10.62	17.98	0.13
	White	13.51	12.59	14.53	
	Other	18.94	13.58	23.80	

^1^log-rank test; * Income categories determined according to Pew Research Reports 2022;**ADI- Area Deprivation Index; ***RUCA-Rural Urban Communicating Area

### Multivariate Cox proportional hazards

**Fig. 2:**
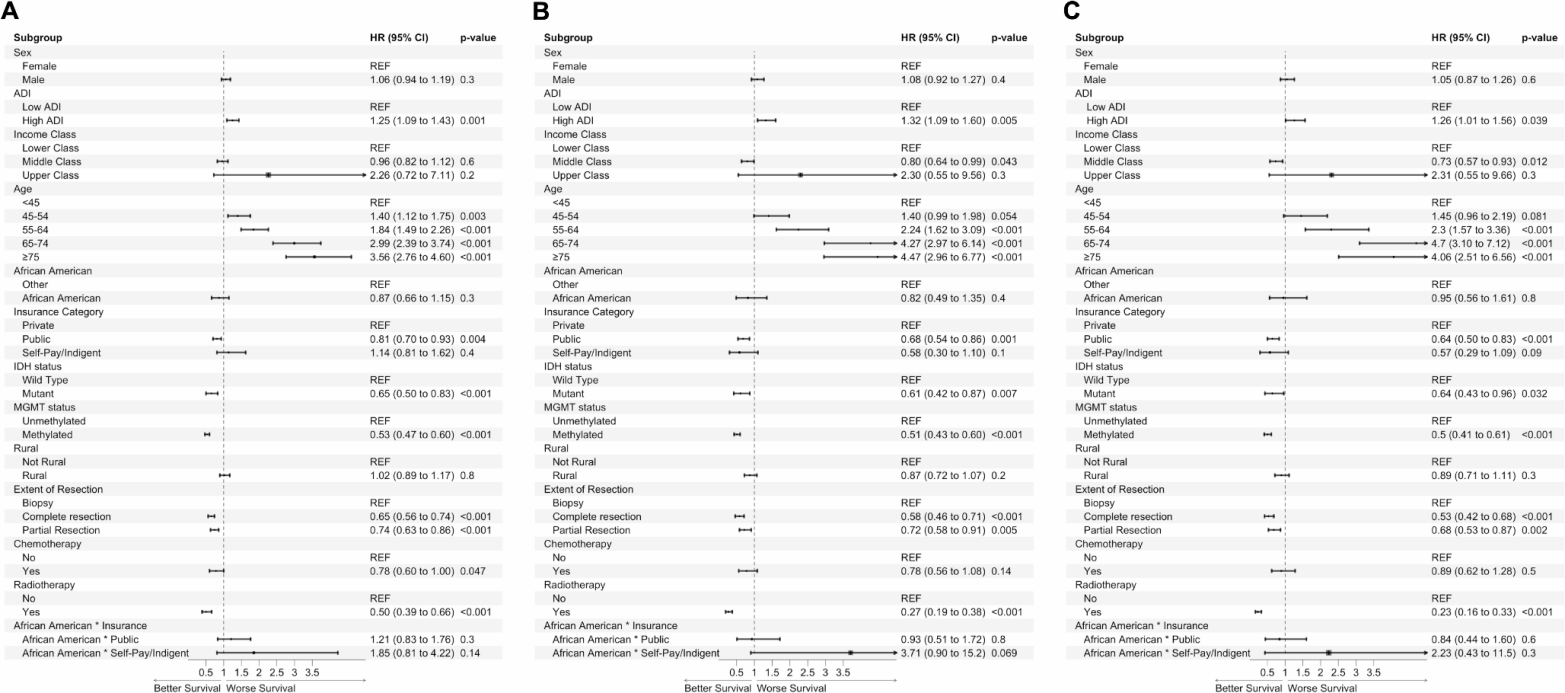
Forest Plot of multivariate cox regression analysis for overall survival **(A)** Imputed cohort **(B)** Complete Case cohort **(C)** Post-2016 WHO guidelines cohort

Using multivariate cox proportional hazards analysis, we identified that patients with high neighborhood disadvantage had worse survival (HR 1.26, 95%CI 1.11–1.43, *p* <.001) compared to patients without. To enhance the robustness of these results, these findings were subsequently replicated in both types of imputation, complete case analysis, and in subgroup analysis of patients diagnosed after the 2016 WHO CNS Guidelines (Supplementary Content, Table 9S). When compared to patients who were in the youngest age group at time of diagnosis (< 45 years), all other age groups had worse survival. Patients who underwent gross total resection had better survival (HR 0.65, 95%CI 0.56–0.74, *p* <.001) compared to patients who underwent partial resections or biopsy. Patients with public insurance had better survival (HR 0.81, 95%CI 0.70–0.93, *p* =.004) compared to patients with private insurance. Patients with IDH mutation had better survival (HR 0.65, 95%CI 0.56–0.74, *p* <.001) compared to those who were IDH-WT. Patients with methylated MGMT had better survival (HR 0.53, 95%CI 0.47–0.61, *p* <.001) when compared to patients with unmethylated MGMT. In all three multivariate models, black race was not found to be associated with worsened survival. Chemotherapy was found to be associated with improved survival in only the MICE model (HR 0.74, 95%CI 0.57–0.95, *p* =.02). Forest plots for this analysis can be found in Fig. 2.

## Discussion

Here we report the first analysis of the effects of neighborhood level socioeconomic status on glioblastoma survival after adjusting for other socioeconomic, clinical, and molecular factors. Our findings strongly suggest that neighborhood deprivation independently predicts survival in patients with glioblastoma and is a potential prognostic marker for patients with glioblastoma.

The role of socioeconomic status in cancer is well known, with many studies highlighting the effect of socioeconomic status on survival of cancer patients. Studies have suggested that income, insurance status, and various other commonly used measures are imperfect proxies of socioeconomic status, varying by region and race. Studies on the association of race and other socioeconomic markers are conflicted as well. Commonly utilized measures of socioeconomic disadvantage in literature can be imperfect proxies for SES [8, 9]. Additionally, social determinants of health play complex roles in determining the health outcomes of patients, and findings may be difficult to generalize on a national level. Thus, the utilization of ADI for neighborhood socioeconomic status represents an advancement in understanding the prognostic effect of patient SES on glioblastoma survival by utilizing a nationally standardized, multifactorial measure of patient socioeconomic disadvantage. 

In this study, we report the effect of neighborhood disadvantage on survival in patients with glioblastoma in the largest single institution cohort to date. Neighborhood socioeconomic disadvantage, as captured by ADI, has emerged as a preferred metric for capturing socioeconomic status in many fields of medicine [11, 18, 19]. However, it has been under-utilized in neuro-oncology and neurosurgery. We observed that high neighborhood disadvantage is independently associated with worse survival (HR 1.25, 95% CI 1.09–1.43, *p* <.001) after adjusting for age, race, income, insurance status, IDH status, MGMT methylation, rurality, extent of resection, history of chemotherapy, and history of radiotherapy. There are many potential mechanisms for this observation.

### Access to care

Patients with high neighborhood disadvantage reflect high degrees of socioeconomic disparity, which has been associated with decreased neurosurgical coverage [20]. This is reinforced in our findings, in which we observed that patients with high neighborhood disadvantage were less likely to have undergone gross total resection compared to patients without (Table 2). Analysis by Perla et al. [21] found that patients with high neighborhood disadvantage had lower access to post-operative care in glioblastoma patients, reflecting a disparity in access to neurosurgical care. Similarly, a study conducted by Guidry et al. [22] found that patients with high neighborhood disadvantage were more likely to be lost to follow-up and have an unplanned readmission following emergent surgery for acute subdural hematoma. Barriers in accessing to care would contribute to a worse prognosis for glioblastoma patients, whether due to socioeconomic or geographic disparities. The association of transportation difficulties would potentially be associated with poor glioblastoma outcomes as well, though we adjusted for rurality, and by proxy, distance from institution in our analysis.

### Adherence to treatment

High neighborhood disadvantage is associated with difficulties in adhering to treatment regimens. An analysis conducted by Brown et al. [23] found that patients with high neighborhood disadvantage had increased rates of no-shows in scheduled telehealth visits. A study by Hensley et al. [24] suggested that patients of low socioeconomic status had lower rates of medication adherence. Similar results were found by Wadhwania et al. [25] in children following liver transplantation, where patients with higher neighborhood-level socioeconomic deprivation had lower levels of medication adherence. Neighborhood-level socioeconomic deprivation may capture barriers in patient education, access to care, or a socioeconomic environment that may present difficulties in adhering to treatment regiments [26]. This association in the context of glioblastoma treatment should be further explored in future studies.

### Clinical comorbidities

High neighborhood disadvantage has also been shown to be associated with comorbidities such as uncontrolled diabetes and cardiovascular health. Durfey et al. [27] reported that patients with high neighborhood disadvantage had more and worse controlled chronic comorbidities. Similarly, Kurani et al. [28] identified that diabetes patients with high neighborhood disadvantage received lower-quality care, leading lower significantly lower likelihood of acceptable HbA1C levels, blood pressure, and lipid levels. Additionally, they identified that increased ADI was also associated with smoking. Lindner et al. [29] also found that ADI was associated with lower glycemic control in patients with Type I Diabetes Mellites (DMI) and higher risk of diabetic ketoacidosis. This was also found by Rodriguez et al. [30], who identified an increased incidence of cardiovascular comorbidities in patients with high neighborhood disadvantage. Worsened comorbidity status could result in worse tolerance of the challenging and taxing course of treatment that is the current standard of care for glioblastoma patients, leading to earlier mortality, which is supported in analysis by Carr et al. [31]

### Health literacy

High neighborhood disadvantage has also been associated with lower levels of health literacy as well. Knighton et al. [32] found that low health literacy was significantly associated with a higher ADI. The relationship between low health literacy and poor health outcomes is well-established in the literature. Poor health literacy may contribute to delays in presentation, lower rates of follow-up care, and lower rates of adherence to treatment regimens [33, 34]. However, the association of health literacy and glioblastoma survival should be investigated in future studies to better understand this mechanism.

### Delayed care

Greater degrees of neighborhood disadvantage could result in delayed initiation of care of glioblastoma, leading to delayed detection and a worse prognosis. Areas of high neighborhood disadvantage generally have lower primary care coverage, and these socioeconomically disadvantaged patients are less likely to be insured and have regular primary care providers (PCPs); this would lead to delays in diagnosis and treatment of glioblastoma in patients with high neighborhood disadvantage and potentially lead to worse outcomes [35]. The association of low socioeconomic and lack of oncological and neurosurgical care is also well established [20, 36, 37]. This is supported by analysis by Aguirre et al. [38] who reported that patients with high neighborhood disadvantage had higher WHO tumor grades at presentation. Delay in cancer care for socioeconomically disadvantaged patients has also been identified in previous literature as well. In an analysis conducted by Ahmad et al. [39], patients of public insurance, and racially minoritized patients were more likely to encounter delays in initiation of treatment for anal squamous cell carcinoma. This is supported in part by our observation that patients with high deprivation were less likely to receive total resections, suggesting a potentially more advanced tumor progression at time of presentation. Additionally, these patients were also less likely to receive radiotherapy, which could potentially be attributed by greater rates of selecting less aggressive treatment regimens due to advanced tumor progression at time of diagnosis, though this difference was not statistically significant (Table 1).

### Interventions

Utilization of neighborhood-level deprivation offers several avenues for systematic interventions to ameliorate disparities in survival. ADI captures patients who may have lower levels of education, income and employment, issues with housing, and disparate housing conditions such as lack of access to internet or phones. Thus, interventions may be designed to address each aspect of neighborhood deprivation captured by ADI (Table 3).

**Table 3:** Components of ADI

Education	Income	Housing	Household characteristics
% Population aged 25 years or older with less than 9 years of education % Population aged 25 years or older with at least a high school diploma % Employed population aged 16 years or older in white-collar occupations	Median family income in US dollars Income disparity % Families below federal poverty level % Population below 150% of federal poverty level % Civilian labor force population aged 16 years and older who are unemployed	Median home value in US dollars Median gross rent in US dollars Median monthly mortgage in US dollars % Owner-occupied housing units % Occupied housing units without complete plumbing	% Single-parent households with children younger than 18 % Households without a motor vehicle % Households without a telephone % Households with more than 1 person per room

To address patients with low educational attainment and health literacy, increased outreach and public education programs should be put in place in communities with high neighborhood deprivation to allow for potentially earlier detection, improved decision-making, and better overall management of disease progression [40]. Assistance with access to primary care and preventative services, enrollment in public food assistance programs and other community programs have also been shown to be of use in improving health outcomes and may be an effective intervention for patients with high neighborhood deprivation [41, 42]. To address the patients with disadvantaged housing characteristics, implementation of medical-legal collaborations and assistance in accessing public housing unites may help address inequitable housing conditions and improve outcomes for patients as well [43, 44]. Assistance through low-cost/free internet access programs, as well as transportation assistance programs may help decrease rates of follow-up loss and improve survival in patients who struggle with low household characteristics [45–48].

Patients with high neighborhood disadvantage may encounter delays of care for glioblastoma, contributing to a worse overall prognosis. Future studies should investigate the relationship between neighborhood socioeconomic status and time to presentation or degree of glioblastoma progression at initial evaluation to better understand this mechanism.

### Limitations

Our study is limited by its retrospective, single center design. New revised 2021 WHO Central Nervous System (CNS) Tumor guidelines categorize IDH mutant, grade IV astrocytomas as a separate entity from glioblastoma. All IDH-mutant tumors were still included in this cohort to understand the socioeconomic disparities that exist in high grade glioma care. However, we controlled for IDH status in our analysis. There was missing data for IDH status and MGMT methylation status in our cohort, largely due to changing patterns of practice and the diagnosis and treatment of patients prior to the adoption of 2016 WHO CNS tumor guidelines. Because of this, we can reasonably suspected that missing data patterns likely met criteria for missing-at-random (MAR), thus justifying the usage of multiple imputations even at higher proportions [49, 50]. Furthermore, two different methods of imputation, complete case analysis, and a separate analysis using only patients diagnosed after the 2016 WHO CNS guidelines were consistent, reinforcing the robustness of our findings. Our study also does not consider quality of life and functional metrics, which may be associated with survival and may be considered as future avenues of study. Though ADI is a highly validated measure of socioeconomic status in medicine, it may fail to completely capture other aspects of a patient’s socioeconomic status. Patient addresses from a single time point may fail to capture potential changes in residence. However, these addresses still represent the best capture of relative socioeconomic status, as represented by neighborhood disparity. Therefore, the address remains the best effort in identifying neighborhood socioeconomic status as a prognostic factor for glioblastoma survival.

## Conclusion

In this study, we have validated neighborhood disadvantage as a prognostic marker for overall survival in glioblastoma. Utilization of neighborhood disadvantage, captured by ADI, allows more nuanced and granular understanding of socioeconomic status in patients with glioblastoma. Patients with high neighborhood disadvantage should be considered at higher risk of poor outcomes and received additional counseling by an interdisciplinary team. Future studies should seek to further validate the effect of neighborhood disadvantage on glioblastoma outcomes in a multi-center cohort and to identify interventions to remedy this disparity.

## Electronic supplementary material

Below is the link to the electronic supplementary material.

Supplementary Material 1

## Data Availability

Data is available upon reasonable request.

## Abbreviations

ADI: Area Deprivation Index
ERS: Economic Research Service
FIPS: Federal Information Processing Standards
HRSA: Health Resources and Services Administration

## Declarations

### Previous presentations

This work was previously presented at the 2024 Congress of Neurological Surgeons in Houston, Texas and received the 2024 CNS Foundation Diversity, Equity, and Inclusion Abstract Award.
