# Supplementary material for: The association of neighborhood-level deprivation with glioblastoma outcomes: a single center cohort study
Source: J Neurooncol. 2025 Apr 7;173(2):457–67. doi: 10.1007/s11060-025-05002-3 (PMC12106151; doi:10.1007/s11060-025-05002-3)
Supplement: Supplementary file 1 — Supplementary Material 1 [file 11060_2025_5002_MOESM1_ESM.docx]

Supplementary Content:

**Supplementary Methods:**

*Defining Variables*

Insurance status was retrieved at time of diagnosis and categorized as uninsured/self-pay, public (including Medicaid and Medicare-only coverage), and private insurance. Using patient zip codes, we estimated distance to institution in miles through methods detailed by the National Ambulatory Medical Care Survey (NAMCS).^1^ Estimated median household was retrieved from the US Census Bureau 5-year estimated household income in 2022 inflation-adjusted dollars.^2^ Income classes were divided according to the Pew Research Center reports for 2022.^3^ Rural Urban Commuting Area (RUCA) codes were categorized in accordance Economic Research Service (ERS) of the United States Department of Agriculture (USDA).^3^ RUCA classifies U.S. census tracts using measures of population density, urbanization, and daily commuting into four main categories (Metropolitan, Micropolitan, Small town and Rural).

*Statistical Analysis*

Assumptions for each test was assessed, multivariate cox proportional hazards models were utilized to assess the association of clinical variables and socioeconomic variables with OS. Schoenfeld’s residuals were used in order to assess assumptions of proportionality (Supplementary Content, Table 1S, Figure 1S).^4^

There was a high degree of missing values for MGMT methylation (39%) and IDH mutation (33%) status. We assumed data was missing at random (MAR) due to inconsistent testing before the release of the 2016 WHO Guidelines on Tumors of the Central Nervous System.^5^ We performed multiple imputations using the random forest imputations method and multiple imputations with chained equations (MICE) for missing values of MGMT status and IDH status with 50 iterations.^6^ Convergence diagrams were plotted for each method. Imputation variance and pooled models were assessed for each imputation method to ensure agreement after imputation. Multiple imputation diagnostics and variance analysis can be found in the supplement.

**Table 1S**. Schoenfeld’s residuals for Multivariate Cox Regression Models

| Random Forest Model | chisq | df | p |
| --- | --- | --- | --- |
| Sex | 1.249 | 1 | 0.264 |
| high_ADI | 0.605 | 1 | 0.437 |
| income_class | 6.183 | 2 | 0.045 |
| age_cat | 24.65 | 4 | 5.90E-05 |
| african_american | 0.795 | 1 | 0.373 |
| insurance_cat | 6.908 | 2 | 0.032 |
| IDH-Mut | 1.174 | 1 | 0.279 |
| MGMT-methylated | 1.271 | 1 | 0.26 |
| rural | 1.331 | 1 | 0.249 |
| surgical_procedure | 42.627 | 2 | 5.50E-10 |
| chemotherapy | 45.183 | 1 | 1.80E-11 |
| radiotherapy | 52.136 | 1 | 5.20E-13 |
| african_american:insurance_cat | 0.194 | 2 | 0.907 |
| GLOBAL | 99.087 | 20 | 1.80E-12 |

| MICE | chisq | df | p |
| --- | --- | --- | --- |
| Sex | 0.6258 | 1 | 0.429 |
| high_ADI | 0.996 | 1 | 0.318 |
| income_class | 5.7912 | 2 | 0.055 |
| age_cat | 30.1305 | 4 | 4.60E-06 |
| african_american | 0.2559 | 1 | 0.613 |
| insurance_cat | 7.3515 | 2 | 0.025 |
| IDH-Mut | 0.0387 | 1 | 0.844 |
| MGMT-methylated | 1.8026 | 1 | 0.179 |
| rural | 2.0546 | 1 | 0.152 |
| surgical_procedure | 36.5169 | 2 | 1.20E-08 |
| chemotherapy | 46.4383 | 1 | 9.50E-12 |
| radiotherapy | 58.5788 | 1 | 2.00E-14 |
| african_american:insurance_cat | 0.4117 | 2 | 0.814 |
| GLOBAL | 101.3728 | 20 | 7.10E-13 |

| Complete Case analysis | chisq | df | p |
| --- | --- | --- | --- |
| Sex | 4.66E-05 | 1 | 0.99455 |
| high_ADI | 1.45E+00 | 1 | 0.22853 |
| income_class | 1.57E+00 | 2 | 0.45529 |
| age_cat | 7.45E+00 | 4 | 0.11384 |
| african_american | 1.22E-01 | 1 | 0.72695 |
| insurance_cat | 9.17E+00 | 2 | 0.01019 |
| IDH-Mut | 5.63E-01 | 1 | 0.45317 |
| MGMT-methylated | 2.41E-01 | 1 | 0.62328 |
| rural | 1.55E-01 | 1 | 0.69411 |
| surgical_procedure | 3.61E+00 | 2 | 0.16483 |
| chemotherapy | 1.31E+01 | 1 | 0.00029 |
| radiotherapy | 1.66E+01 | 1 | 4.50E-05 |
| african_american:insurance_cat | 3.83E-01 | 2 | 0.82568 |
| GLOBAL | 3.53E+01 | 20 | 0.01846 |

| Post WHO 2016 | chisq | df | p |
| --- | --- | --- | --- |
| Sex | 0.7883 | 1 | 0.37461 |
| high_ADI | 4.5163 | 1 | 0.03357 |
| income_class | 0.9205 | 2 | 0.63114 |
| age_cat | 5.2903 | 4 | 0.25879 |
| african_american | 0.3413 | 1 | 0.55905 |
| insurance_cat | 11.2544 | 2 | 0.0036 |
| IDH-Mut | 1.5516 | 1 | 0.2129 |
| MGMT-methylated | 1.2183 | 1 | 0.26969 |
| rural | 0.0155 | 1 | 0.90096 |
| surgical_procedure | 4.7607 | 2 | 0.09252 |
| chemotherapy | 19.0805 | 1 | 1.30E-05 |
| radiotherapy | 21.7255 | 1 | 3.10E-06 |
| african_american:insurance_cat | 0.0318 | 2 | 0.98421 |
| GLOBAL | 52.1994 | 20 | 0.00011 |

**Table 2S.** Pooled Cox Regression for Random Forest Imputation

| **Pooled cox Proportional Hazards Analysis** | |  |  |  |  |
| --- | --- | --- | --- | --- | --- |
| **Term** | **Estimate** | **Std. Error** | **Statistic** | **DF** | **P-Value** |
| SexMale | 0.05291026 | 0.06053455 | 0.87405069 | 980.36915 | 3.82E-01 |
| ADI_NATRANK > 75TRUE | 0.22098405 | 0.06959691 | 3.17519933 | 713.3921 | 1.56E-03 |
| age_cat≥75 | 1.22969736 | 0.14392967 | 8.54373783 | 612.84503 | 1.03E-16 |
| age_cat45-54 | 0.35310925 | 0.12447194 | 2.83685815 | 721.90197 | 4.68E-03 |
| age_cat55-64 | 0.59977806 | 0.11820604 | 5.0740053 | 559.64431 | 5.31E-07 |
| age_cat65-74 | 1.06730217 | 0.12972426 | 8.22746788 | 475.41329 | 1.85E-15 |
| african_american | -0.1757958 | 0.14800804 | -1.1877451 | 968.95253 | 2.35E-01 |
| insurance_catPublic | -0.1811729 | 0.08005416 | -2.2631298 | 780.87515 | 2.39E-02 |
| insurance_catSelf-Pay/Indigent | 0.12527752 | 0.18184071 | 0.68894097 | 1106.19665 | 4.91E-01 |
| IDH-Mut | -0.4578104 | 0.15307009 | -2.9908548 | 328.70944 | 2.99E-03 |
| MGMT-methylated | -0.5924692 | 0.10245693 | -5.782617 | 98.59674 | 8.67E-08 |
| rural | 0.00258907 | 0.07579693 | 0.03415801 | 630.85091 | 9.73E-01 |
| surgical_procedureComplete resection | -0.4668157 | 0.07600114 | -6.1422201 | 774.1259 | 1.30E-09 |
| surgical_procedurePartial Resection | -0.3478059 | 0.08527324 | -4.0787228 | 879.79046 | 4.94E-05 |
| chemotherapyYes | -0.2197899 | 0.13247789 | -1.6590684 | 1086.18591 | 9.74E-02 |
| radiotherapyYes | -0.6812608 | 0.14479848 | -4.7048889 | 898.78694 | 2.94E-06 |
| african_american: Public | 0.22061071 | 0.19828863 | 1.11257366 | 1025.70274 | 2.66E-01 |
| african_american: Self Pay/Indigent | 0.77455086 | 0.43246552 | 1.79101181 | 1050.12313 | 7.36E-02 |

**Table 3S.** Pooled Cox Regression for MICE

| **Pooled cox Proportional Hazards Analysis** | | | |  |  |
| --- | --- | --- | --- | --- | --- |
| **Term** | **Estimate** | **Std. Error** | **Statistic** | **df** | **p.value** |
| SexMale | 0.05219202 | 0.06247096 | 0.83546063 | 763.0443 | 0.4037199 |
| ADI_NATRANK > 75TRUE | 0.23009093 | 0.06973499 | 3.29509483 | 710.1873 | 0.0016901 |
| age_cat75 | 1.17297999 | 0.15067451 | 7.78486003 | 435.7822 | 5.13E-14 |
| age_cat45-54 | 0.30094866 | 0.12092943 | 2.48863035 | 870.4213 | 0.01300956 |
| age_cat55-64 | 0.53197471 | 0.11355937 | 4.68455142 | 758.7342 | 3.32E-06 |
| age_cat65-74 | 0.99245525 | 0.12706796 | 7.81042878 | 558.7545 | 2.84E-14 |
| african_american | -0.2113588 | 0.15344535 | -1.3774206 | 724.764 | 0.1688075 |
| insurance_catPublic | -0.2122988 | 0.08505148 | -2.6355672 | 720.4628 | 0.00858037 |
| insurance_catSelf-Pay/Indigent | 0.08587514 | 0.18142649 | 0.47333297 | 1120.2047 | 0.6360679 |
| IDH-Mut | -0.572072 | 0.16063905 | -3.5612264 | 122.0569 | 5.27E-04 |
| MGMT-methylated | -0.5806464 | 0.1019292 | -5.6965656 | 102.0117 | 1.19E-07 |
| rural | 0.00460526 | 0.00755304 | 0.6097227 | 659.1512 | 0.513998 |
| surgical_procedureComplete resection | -0.4860164 | 0.07483761 | -6.49428 | 906.6632 | 1.37E-10 |
| surgical_procedurePartial Resection | -0.3522226 | 0.08585348 | -4.102601 | 827.322 | 4.49E-05 |
| chemotherapyYes | -0.2243171 | 0.13635704 | -1.6450717 | 932.111 | 0.1002922 |
| radiotherapyYes | -0.6748838 | 0.14709469 | -4.5880907 | 835.5717 | 5.16E-06 |
| african_american: Public | 0.26464671 | 0.20062459 | 1.31811402 | 940.5318 | 0.1874521 |
| african_american: Self Pay/Indigent | 0.76754055 | 0.43159912 | 1.7783645 | 1062.4099 | 0.07562993 |

**Table 4S.** Random Forest Imputation Diagnostics

| **Term** | **m** | **Estimate** | **Within** | **Between** | **Total** | **dfcom** | **df** | **RIV** | **Lambda** | **FMI** | **Relative Efficiency** |
| --- | --- | --- | --- | --- | --- | --- | --- | --- | --- | --- | --- |
| SexMale | 50 | 0.05291026 | 0.00338909 | 0.00026994 | 0.00366443 | 1197 | 980.36915 | 0.08124344 | 0.07513889 | 0.0770199 | 0.998461971 |
| ADI_NATRANK > 75TRUE | 50 | 0.22098405 | 0.04145556 | 0.00684484 | 0.04843729 | 1197 | 713.3921 | 0.16841488 | 0.14413962 | 0.14652899 | 0.997077983 |
| age_cat75 | 50 | 1.22969736 | 0.17109317 | 0.03535719 | 0.2071575 | 1197 | 612.84503 | 0.21078769 | 0.17409137 | 0.17677357 | 0.996476984 |
| age_cat45-54 | 50 | 0.35310925 | 0.01329678 | 0.02153417 | 0.01549327 | 1197 | 721.90197 | 0.16518923 | 0.1417703 | 0.14413815 | 0.997125523 |
| age_cat55-64 | 50 | 0.59977806 | 0.01129167 | 0.00262843 | 0.01397267 | 1197 | 559.64431 | 0.237432 | 0.19187479 | 0.19474739 | 0.996120164 |
| age_cat65-74 | 50 | 1.06730217 | 0.01305713 | 0.03697307 | 0.01682838 | 1197 | 475.41329 | 0.28882709 | 0.22410073 | 0.22734436 | 0.995473693 |
| african_american | 50 | -0.1757958 | 0.20196271 | 0.01676579 | 0.21906381 | 1197 | 968.95253 | 0.08467456 | 0.07806448 | 0.07996156 | 0.998403322 |
| insurance_catPublic | 50 | -0.1811729 | 0.00560204 | 0.00790811 | 0.00640867 | 1197 | 780.87515 | 0.1439881 | 0.12586503 | 0.12809532 | 0.99744464 |
| insurance_catSelf-Pay/Indigent | 50 | 0.12527752 | 0.03174782 | 0.01292382 | 0.03366045 | 1197 | 1106.19665 | 0.04152191 | 0.03986658 | 0.0415978 | 0.999168736 |
| IDH-Mut | 50 | -0.4578104 | 0.16384521 | 0.06907776 | 0.23430452 | 1197 | 328.70944 | 0.43003585 | 0.30071683 | 0.30493307 | 0.993938307 |
| MGMT-methylated | 50 | -0.5924692 | 0.03956975 | 0.06412203 | 0.10497422 | 1197 | 98.59674 | 1.65289065 | 0.62305269 | 0.63047315 | 0.987547556 |
| rural | 50 | 0.00258907 | 0.00477759 | 0.00094862 | 0.00574518 | 1197 | 630.85091 | 0.20252652 | 0.16841751 | 0.17104141 | 0.996590834 |
| surgical_procedureComplete resection | 50 | -0.4668157 | 0.00503887 | 0.00722845 | 0.00576173 | 1197 | 774.1259 | 0.14632289 | 0.12764544 | 0.12989052 | 0.997408921 |
| surgical_procedurePartial Resection | 50 | -0.3478059 | 0.00654046 | 0.00716728 | 0.00727153 | 1197 | 879.79046 | 0.11177534 | 0.1005377 | 0.10257548 | 0.99795269 |
| chemotherapyYes | 50 | -0.2197899 | 0.01674117 | 0.00793349 | 0.01755039 | 1197 | 1086.18591 | 0.0483369 | 0.04610817 | 0.04785974 | 0.999043721 |
| radiotherapyYes | 50 | -0.6812608 | 0.0189587 | 0.00196853 | 0.0209666 | 1197 | 898.78694 | 0.10590902 | 0.09576648 | 0.09777191 | 0.998048378 |
| african_american: Public Insurance | 50 | 0.22061071 | 0.03683174 | 0.00243789 | 0.03931838 | 1197 | 1025.70274 | 0.06751365 | 0.06324383 | 0.06506507 | 0.99870039 |
| african_american: Indigent | 50 | 0.77455086 | 0.1764483 | 0.01037071 | 0.18702643 | 1197 | 1050.12313 | 0.05995029 | 0.05655953 | 0.05835123 | 0.998834336 |

**Table 5S.** MICE Diagnostics

| SexMale | **m** | **Estimate** | **Within** | **Between** | **Total** | **dfcom** | **df** | **RIV** | **Lambda** | **FMI** | **Relative Efficiency** |
| --- | --- | --- | --- | --- | --- | --- | --- | --- | --- | --- | --- |
| ADI_NATRANK > 75TRUE | 50 | 0.05219202 | 0.00339298 | 0.00049965 | 0.00390262 | 1197 | 763.0443 | 0.15020393 | 0.13058895 | 0.13285883 | 0.99734987 |
| age_cat75 | 50 | 0.23009093 | 0.00415766 | 0.00069148 | 0.00486297 | 1197 | 710.1873 | 0.16964192 | 0.14503748 | 0.14743506 | 0.99705997 |
| age_cat45-54 | 50 | 1.17297999 | 0.01721763 | 0.05377628 | 0.02270281 | 1197 | 435.7822 | 0.31857937 | 0.24160803 | 0.24506484 | 0.99512261 |
| age_cat55-64 | 50 | 0.30094866 | 0.01311919 | 0.00147523 | 0.01462393 | 1197 | 870.4213 | 0.11469748 | 0.10289561 | 0.10494984 | 0.9979054 |
| age_cat65-74 | 50 | 0.53197471 | 0.01119683 | 0.00166558 | 0.01289573 | 1197 | 758.7342 | 0.15173007 | 0.131741 | 0.13402069 | 0.99732675 |
| african_american | 50 | 0.99245525 | 0.01304317 | 0.00304225 | 0.01614627 | 1197 | 558.7545 | 0.23790994 | 0.19218679 | 0.19506283 | 0.9961139 |
| insurance_catPublic | 50 | -0.2113588 | 0.02022608 | 0.03254312 | 0.02354548 | 1197 | 724.764 | 1.25211692 | 0.55597332 | 0.56307452 | 0.98886392 |
| insurance_catSelf-Pay/Indigent | 50 | -0.2122988 | 0.00556607 | 0.00090439 | 0.00648854 | 1197 | 720.4628 | 1.57781949 | 0.61207524 | 0.61946346 | 0.98776235 |
| IDH-Mut | 50 | 0.08587514 | 0.03175609 | 0.00113674 | 0.03291557 | 1197 | 1120.2047 | 0.19019022 | 0.15979817 | 0.16233596 | 0.99676379 |
| MGMT-methylated | 50 | -0.572072 | 0.01145807 | 0.14065528 | 0.02580491 | 1197 | 122.0569 | 0.16573148 | 0.14216952 | 0.14454097 | 0.99711751 |
| rural | 50 | -0.5806464 | 0.00403037 | 0.0062345 | 0.01038956 | 1197 | 102.0117 | 0.03651194 | 0.03522578 | 0.03694368 | 0.99926167 |
| surgical_procedureComplete resection | 50 | 0.00460526 | 0.00479322 | 0.00089375 | 0.00570484 | 1197 | 659.1512 | 0.10349677 | 0.09378983 | 0.09578223 | 0.99808802 |
| surgical_procedurePartial Resection | 50 | -0.4860164 | 0.00507538 | 0.00051499 | 0.00560067 | 1197 | 906.6632 | 0.12844676 | 0.11382616 | 0.11596069 | 0.99768615 |
| chemotherapyYes | 50 | -0.3522226 | 0.00653183 | 0.00082254 | 0.00737082 | 1197 | 827.322 | 0.09576555 | 0.08739602 | 0.08934788 | 0.99821623 |
| radiotherapyYes | 50 | -0.2243171 | 0.01696827 | 0.00159311 | 0.01859324 | 1197 | 932.111 | 0.1257711 | 0.11171996 | 0.11383851 | 0.9977284 |
| african_american: Public | 50 | -0.6748838 | 0.01921958 | 0.00236987 | 0.02163685 | 1197 | 835.5717 | 0.16411476 | 0.14097816 | 0.14333888 | 0.99714142 |
| african_american: self Pay/Indigent | 50 | 0.26464671 | 0.03681793 | 0.00336499 | 0.04025023 | 1197 | 940.5318 | 0.08150116 | 0.07535929 | 0.07724149 | 0.99845755 |
|  | 50 | 0.76754055 | 0.17638825 | 0.00969563 | 0.1862778 | 1197 | 1062.4099 | 0.0506669 | 0.05309029 | 0.05486784 | 0.99890385 |

**Table 6S.** Patient Demographics

| **Characteristic** | **N = 1,464***^1^* |
| --- | --- |
| Age at Diagnosis (years) | 60 ± 14 |
| < 45 | 211 (14%) |
| ≥75 | 185 (13%) |
| 45-54 | 231 (16%) |
| 55-64 | 392 (27%) |
| 65-74 | 445 (30%) |
| Sex |  |
| Female | 648 (44%) |
| Male | 816 (56%) |
| Race |  |
| White | 1,224 (84%) |
| Black | 155 (11%) |
| Other | 85 (5.8%) |
| Married | 1,033 (71%) |
| Insurance Status |  |
| Indigent/Self Pay | 51 (3.5%) |
| Medicaid | 111 (7.6%) |
| Medicare | 590 (40%) |
| Private | 712 (49%) |
| Median Household Income (USD) | 50,055 (42,285, 59,264) |
| Vital Status |  |
| Alive | 249 (17%) |
| Deceased | 1,215 (83%) |
| IDH status |  |
| IDH-Mut | 92 (6.3%) |
| IDH-WT | 890 (61%) |
| Unknown | 482 (33%) |
| MGMT status |  |
| Methylated | 344 (23%) |
| Unknown | 576 (39%) |
| Unmethylated | 544 (37%) |
| Extent of Resection |  |
| Biopsy | 430 (29%) |
| Complete resection | 671 (46%) |
| Partial Resection | 363 (25%) |
| History of Radiotherapy | 1,235 (84%) |
| History of Chemotherapy | 1,219 (83%) |
| ADI National Percentile Rank | 66 (46, 84) |
| RUCA |  |
| Metropolitan | 1,062 (73%) |
| Micropolitan | 225 (15%) |
| Rural | 51 (3.5%) |
| Small Town | 126 (8.6%) |
| Distance from Institution |  |
| <60 | 617 (42%) |
| ≥200 | 149 (10%) |
| 60-200 | 698 (48%) |
| Elixhauser Comorbidity Index | 12 (7, 20) |
| Missing | 160 |
| Overall Survival (months) | 13.78 (12.99 - 14.76) |
| *^1^* n (%); Median (IQR)*;* Mean ± SD |  |

**Table 7S.** Comorbidity Burden

| **Characteristic** | **Low ADI** | **High ADI** | **p-value***^2^* |
| --- | --- | --- | --- |
|  | N = 912*^1^* | N = 552*^1^* |  |
| Elixhauser Comorbidity Score | 12 (8, 20) | 13 (4, 20) | 0.2 |
| Congestive Heart Failure | 41 (5.0%) | 26 (5.4%) | 0.8 |
| Cardiac Arrhythmias | 171 (21%) | 92 (19%) | 0.4 |
| Valvular Disease | 36 (4.4%) | 14 (2.9%) | 0.2 |
| Pulmonary Circulation Disorder | 43 (5.2%) | 35 (7.2%) | 0.14 |
| Peripheral Vascular Disorder | 53 (6.5%) | 36 (7.4%) | 0.5 |
| Hypertension, uncomplicated | 392 (48%) | 258 (53%) | 0.055 |
| Hypertension, complicated | 51 (6.2%) | 25 (5.2%) | 0.4 |
| Paralysis | 170 (21%) | 112 (23%) | 0.3 |
| Other neurological disorder | 540 (66%) | 293 (61%) | 0.053 |
| Chronic Pulmonary Disease | 82 (10%) | 47 (9.7%) | 0.9 |
| Diabetes, uncomplicated | 51 (6.2%) | 40 (8.3%) | 0.2 |
| Diabetes, complicated | 68 (8.3%) | 51 (11%) | 0.2 |
| Hypothyroidism | 92 (11%) | 55 (11%) | >0.9 |
| Renal Failure | 31 (3.8%) | 20 (4.1%) | 0.8 |
| Liver Disease | 58 (7.1%) | 22 (4.5%) | 0.066 |
| Peptic Ulcer Disease | 3 (0.4%) | 2 (0.4%) | >0.9 |
| AIDS/HIV | 0 (0%) | 0 (0%) |  |
| Lymphoma | 8 (1.0%) | 5 (1.0%) | >0.9 |
| Metastatic Cancer | 103 (13%) | 44 (9.1%) | 0.056 |
| Solid tumor, without metastasis | 749 (91%) | 442 (91%) | >0.9 |
| Rheumatoid arthritis/collaged vascular disease | 22 (2.7%) | 8 (1.7%) | 0.2 |
| Coagulopathy | 115 (14%) | 65 (13%) | 0.8 |
| Obesity | 164 (20%) | 94 (19%) | 0.8 |
| Weight Loss | 46 (5.6%) | 33 (6.8%) | 0.4 |
| Fluid and electrolyte disorders | 333 (41%) | 210 (43%) | 0.3 |
| Blood Loss Anemia | 2 (0.2%) | 0 (0%) | 0.5 |
| Deficiency Anemia | 24 (2.9%) | 17 (3.5%) | 0.6 |
| Alcohol Abuse | 21 (2.6%) | 11 (2.3%) | 0.7 |
| Drug Abuse | 20 (2.4%) | 15 (3.1%) | 0.5 |
| Psychoses | 13 (1.6%) | 13 (2.7%) | 0.2 |
| Depression | 185 (23%) | 124 (26%) | 0.2 |
| *^2^* Pearson’s Chi-squared test; Fisher’s exact test |  |  |  |

Table 8S. Survival analysis

|  |  |  |  |  |  |
| --- | --- | --- | --- | --- | --- |
|  | **Level** | **Median survival** | **CI lower** | **CI upper** | **p-value**^1^ |
| IDH status | IDH-Mut | 33.57 | 27.32 | 41.79 | <.001 |
|  | IDH-WT | 13.08 | 12.09 | 14.14 |  |
|  | Unknown | 13.12 | 11.80 | 14.89 |  |
| MGMT Status | Methylated | 21.14 | 18.35 | 23.08 | <.001 |
|  | Unmethylated | 12.99 | 11.74 | 14.10 |  |
|  | Unknown | 12.26 | 10.49 | 13.48 |  |
| Extent of Resection | Biopsy | 7.43 | 5.85 | 8.84 | <.001 |
|  | Complete resection | 17.03 | 16.14 | 19.04 |  |
|  | Partial Resection | 14.01 | 12.33 | 15.58 |  |
| Chemotherapy | No | 3.68 | 3.12 | 4.67 | <.001 |
|  | Yes | 15.42 | 14.63 | 16.14 |  |
| Radiotherapy | No | 3.22 | 3.02 | 4.04 | <.001 |
|  | Yes | 15.55 | 14.70 | 16.21 |  |
| IDH-WT only | Low ADI | 14.1 | 13.08 | 15.5 | 0.014 |
|  | High ADI | 11.1 | 9.53 | 13.0 |  |

**Table 9S.** Results of Multivariate Cox Regression Analysis

| Random Forest | |  |  | Complete Case Analysis | |  |  | Normal MICE | |  |  | Post WHO Guidelines (2017 to present) | | |  |
| --- | --- | --- | --- | --- | --- | --- | --- | --- | --- | --- | --- | --- | --- | --- | --- |
| **Characteristic** | **HR***^1^* | **95% CI***^1^* | **p-value** | **Characteristic** | **HR***^1^* | **95% CI***^1^* | **p-value** | **Characteristic** | **HR***^1^* | **95% CI***^1^* | **p-value** | **Characteristic** | **HR***^1^* | **95% CI***^1^* | **p-value** |
| Sex |  |  |  | Sex |  |  |  | Sex |  |  |  | Sex |  |  |  |
| Female | — | — |  | Female | — | — |  | Female | — | — |  | Female | — | — |  |
| Male | 1.06 | 0.94, 1.19 | 0.3 | Male | 1.08 | 0.92, 1.27 | 0.4 | Male | 1.03 | 0.92, 1.16 | 0.6 | Male | 1.05 | 0.87, 1.26 | 0.6 |
| ADI |  |  |  | ADI |  |  |  | ADI |  |  |  | ADI |  |  |  |
| Low ADI | — | — |  | Low ADI | — | — |  | Low ADI | — | — |  | Low ADI | — | — |  |
| **High ADI** | **1.25** | **1.09, 1.43** | **0.001** | **High ADI** | **1.32** | **1.09, 1.60** | **0.005** | **High ADI** | **1.21** | **1.06, 1.38** | **0.005** | High ADI | 1.26 | 1.01, 1.56 | 0.039 |
| Income Class | |  |  | Income Class | |  |  | Income Class | |  |  | Income Class | |  |  |
| Lower Class | — | — |  | Lower Class | — | — |  | Lower Class | — | — |  | Lower Class | — | — |  |
| Middle Class | 0.96 | 0.82, 1.12 | 0.6 | Middle Class | 0.8 | 0.64, 0.99 | 0.043 | Middle Class | 0.98 | 0.83, 1.14 | 0.8 | Middle Class | 0.73 | 0.57, 0.93 | 0.012 |
| Upper Class | 2.26 | 0.72, 7.11 | 0.2 | Upper Class | 2.3 | 0.55, 9.56 | 0.3 | Upper Class | 2.2 | 0.70, 6.92 | 0.2 | Upper Class | 2.31 | 0.55, 9.66 | 0.3 |
| Age |  |  |  | Age |  |  |  | Age |  |  |  | Age |  |  |  |
| < 45 | — | — |  | < 45 | — | — |  | < 45 | — | — |  | < 45 | — | — |  |
| 45-54 | 1.4 | 1.12, 1.75 | 0.003 | 45-54 | 1.4 | 0.99, 1.98 | 0.054 | 45-54 | 1.25 | 1.00, 1.57 | 0.05 | 45-54 | 1.45 | 0.96, 2.19 | 0.081 |
| 55-64 | 1.84 | 1.49, 2.26 | <0.001 | 55-64 | 2.24 | 1.62, 3.09 | <0.001 | 55-64 | 1.63 | 1.32, 2.00 | <0.001 | 55-64 | 2.3 | 1.57, 3.36 | <0.001 |
| 65-74 | 2.99 | 2.39, 3.74 | <0.001 | 65-74 | 4.27 | 2.97, 6.14 | <0.001 | 65-74 | 2.44 | 1.95, 3.04 | <0.001 | 65-74 | 4.7 | 3.10, 7.12 | <0.001 |
| ≥75 | 3.56 | 2.76, 4.60 | <0.001 | ≥75 | 4.47 | 2.96, 6.77 | <0.001 | ≥75 | 2.85 | 2.20, 3.68 | <0.001 | ≥75 | 4.06 | 2.51, 6.56 | <0.001 |
| African American | |  |  | African American | |  |  | African American | |  |  | African American | |  |  |
| Other | — | — |  | Other | — | — |  | Other | — | — |  | Other | — | — |  |
| African American | 0.87 | 0.66, 1.15 | 0.3 | African American | 0.82 | 0.49, 1.35 | 0.4 | African American | 0.8 | 0.60, 1.05 | 0.11 | African American | 0.95 | 0.56, 1.61 | 0.8 |
| Insurance Category | |  |  | Insurance Category | |  |  | Insurance Category | |  |  | Insurance Category | |  |  |
| Private | — | — |  | Private | — | — |  | Private | — | — |  | Private | — | — |  |
| Public | 0.81 | 0.70, 0.93 | 0.004 | Public | 0.68 | 0.54, 0.86 | 0.001 | Public | 0.84 | 0.72, 0.97 | 0.016 | Public | 0.64 | 0.50, 0.83 | <0.001 |
| Self-Pay/Indigent | 1.14 | 0.81, 1.62 | 0.4 | Self-Pay/Indigent | 0.58 | 0.30, 1.10 | 0.1 | Self-Pay/Indigent | 1.15 | 0.81, 1.64 | 0.4 | Self-Pay/Indigent | 0.57 | 0.29, 1.09 | 0.09 |
| IDH status | |  |  | IDH status | |  |  | IDH status | |  |  | IDH status | |  |  |
| Wild Type | — | — |  | Wild Type | — | — |  | Wild Type | — | — |  | Wild Type | — | — |  |
| Mutant | 0.65 | 0.50, 0.83 | <0.001 | Mutant | 0.61 | 0.42, 0.87 | 0.007 | Mutant | 0.51 | 0.41, 0.63 | <0.001 | Mutant | 0.64 | 0.43, 0.96 | 0.032 |
| MGMT status | |  |  | MGMT status | |  |  | MGMT status | |  |  | MGMT status | |  |  |
| Unmethylated | — | — |  | Unmethylated | — | — |  | Unmethylated | — | — |  | Unmethylated | — | — |  |
| Methylated | 0.53 | 0.47, 0.60 | <0.001 | Methylated | 0.51 | 0.43, 0.61 | <0.001 | Methylated | 0.59 | 0.52, 0.66 | <0.001 | Methylated | 0.5 | 0.41, 0.61 | <0.001 |
| Rural |  |  |  | Rural |  |  |  | Rural |  |  |  | Rural |  |  |  |
| Not Rural | — | — |  | Not Rural | — | — |  | Not Rural | — | — |  | Not Rural | — | — |  |
| Rural | 1.02 | 0.89, 1.17 | 0.8 | Rural | 0.87 | 0.72, 1.07 | 0.2 | Rural | 0.99 | 0.86, 1.13 | 0.9 | Rural | 0.89 | 0.71, 1.11 | 0.3 |
| Extent of Resection | |  |  | Extent of Resection | |  |  | Extent of Resection | |  |  | Extent of Resection | |  |  |
| Biopsy | — | — |  | Biopsy | — | — |  | Biopsy | — | — |  | Biopsy | — | — |  |
| Complete resection | 0.65 | 0.56, 0.74 | <0.001 | Complete resection | 0.58 | 0.46, 0.71 | <0.001 | Complete resection | 0.63 | 0.55, 0.73 | <0.001 | Complete resection | 0.53 | 0.42, 0.68 | <0.001 |
| Partial Resection | 0.74 | 0.63, 0.86 | <0.001 | Partial Resection | 0.72 | 0.58, 0.91 | 0.005 | Partial Resection | 0.72 | 0.62, 0.85 | <0.001 | Partial Resection | 0.68 | 0.53, 0.87 | 0.002 |
| Chemotherapy | |  |  | Chemotherapy | |  |  | Chemotherapy | |  |  | Chemotherapy | |  |  |
| No | — | — |  | No | — | — |  | No | — | — |  | No | — | — |  |
| Yes | 0.78 | 0.60, 1.00 | 0.047 | Yes | 0.78 | 0.56, 1.08 | 0.14 | Yes | 0.73 | 0.56, 0.94 | 0.016 | Yes | 0.89 | 0.62, 1.28 | 0.5 |
| Radiotherapy | |  |  | Radiotherapy | |  |  | Radiotherapy | |  |  | Radiotherapy | |  |  |
| No | — | — |  | No | — | — |  | No | — | — |  | No | — | — |  |
| Yes | 0.5 | 0.39, 0.66 | <0.001 | Yes | 0.27 | 0.19, 0.38 | <0.001 | Yes | 0.54 | 0.41, 0.71 | <0.001 | Yes | 0.23 | 0.16, 0.33 | <0.001 |
| African American * Insurance | | |  | African American * Insurance | | |  | African American * Insurance | | |  | African American * Insurance | | |  |
| African American * Public | 1.21 | 0.83, 1.76 | 0.3 | African American * Public | 0.93 | 0.51, 1.72 | 0.8 | African American * Public | 1.31 | 0.90, 1.91 | 0.2 | African American * Public | 0.84 | 0.44, 1.60 | 0.6 |
| African American * Self-Pay/Indigent | 1.85 | 0.81, 4.22 | 0.14 | African American * Self-Pay/Indigent | 3.71 | 0.90, 15.2 | 0.069 | African American * Self-Pay/Indigent | 1.92 | 0.84, 4.37 | 0.12 | African American * Self-Pay/Indigent | 2.23 | 0.43, 11.5 | 0.3 |
| *^1^* HR = Hazard Ratio, CI = Confidence Interval | | | | *^1^* HR = Hazard Ratio, CI = Confidence Interval | | | | *^1^* HR = Hazard Ratio, CI = Confidence Interval | | | | *^1^* HR = Hazard Ratio, CI = Confidence Interval | | | |


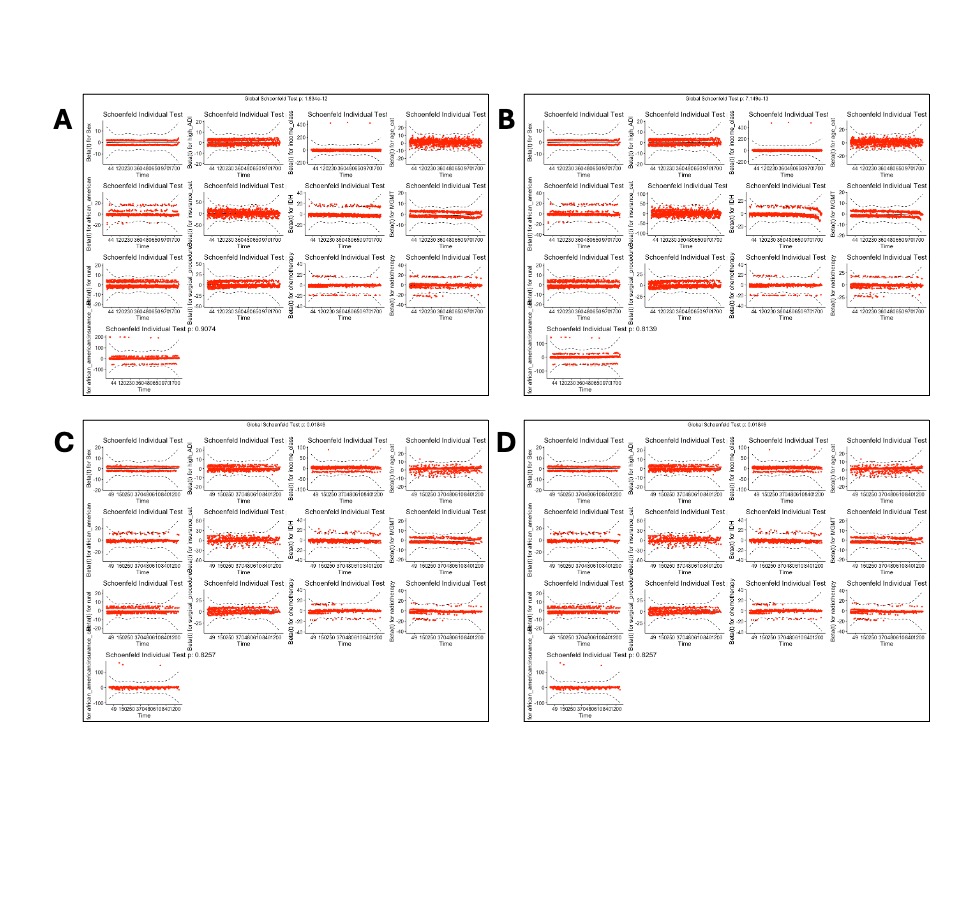


**Figure 1S.** Schoenfeld’s Residuals for **A.** Random Forest imputed **B.** MICE imputed **C.** Complete Case **D.** Post-2016 WHO Guidelines cohorts.


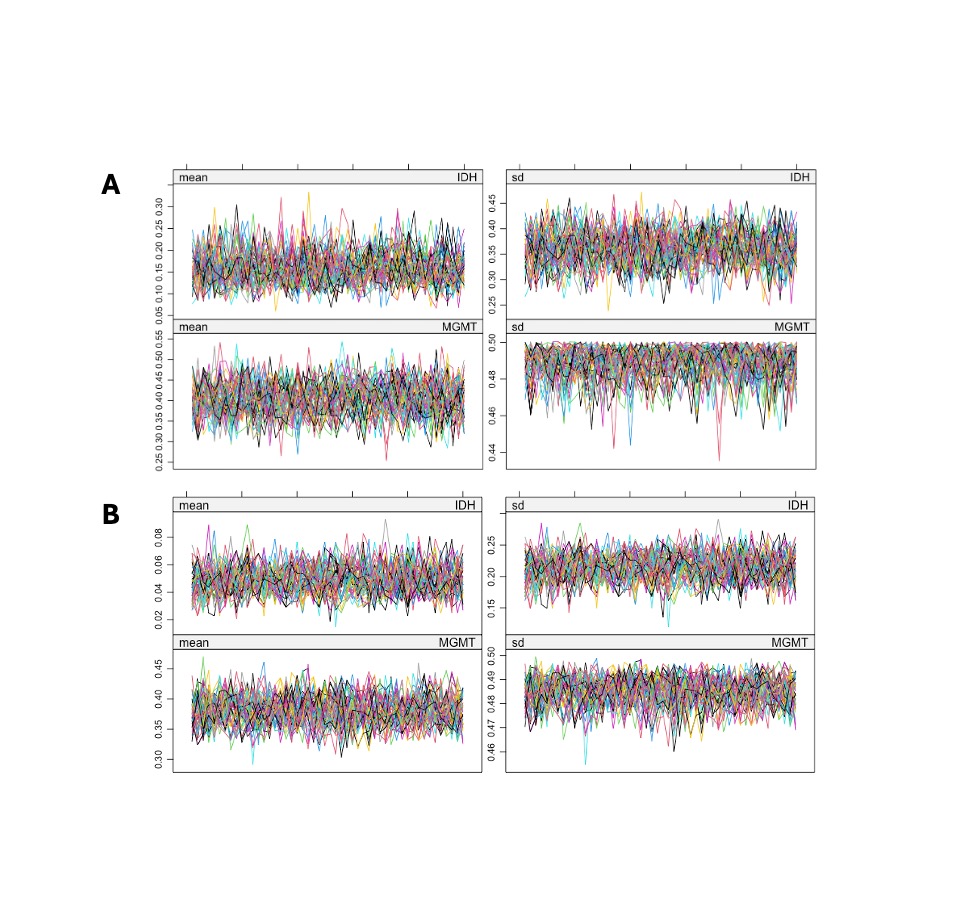


**Figure 2S.** Convergence Plots for **A.** Random Forest imputation **B.** MICE

**Figure 3S.** Kaplan Meier curve for survival in IDH-WT only

1. NAMCS/NHAMCS - Ambulatory Health Care Data Homepage. 2023.

2. Bureau USC. Data from: ACS 5-Year Estimates Subject Tables

Income in the Past 12 Months (in 2022 Inflation-Adjusted Dollars). 2022.

3. Kochhar R, Sechopoulos S. How the American middle class has changed in the past five decades. *Pew Research Center* blog. <https://www.pewresearch.org/short-reads/2022/04/20/how-the-american-middle-class-has-changed-in-the-past-five-decades/>

files/68/how-the-american-middle-class-has-changed-in-the-past-five-decades.html

4. In J, Lee DK. Survival analysis: part II - applied clinical data analysis. *Korean J Anesthesiol*. Oct 2019;72(5):441-457. doi:10.4097/kja.19183

5. Louis DN, Perry A, Reifenberger G, et al. The 2016 World Health Organization Classification of Tumors of the Central Nervous System: a summary. *Acta Neuropathol*. Jun 2016;131(6):803-20. doi:10.1007/s00401-016-1545-1

6. Sterne JAC, White IR, Carlin JB, et al. Multiple imputation for missing data in epidemiological and clinical research: potential and pitfalls. *BMJ*. 2009;338:b2393. doi:10.1136/bmj.b2393
